# Machine-learning prediction of 30-day infection-related hospitalization in advanced CKD

**DOI:** 10.1186/s12879-026-13308-4

**Published:** 2026-05-30

**Authors:** Mu-Chi Chung, Tung-Min Yu, Min-Shian Wang, Ya-Wen Chuang, Ming-Ju Wu, Cheng-Hsu Chen, Chi-Jung Chung

**Affiliations:** 1 Department of Post-Baccalaureate Medicine, College of Medicine, National Chung Hsing University, Taichung, Taiwan; 2 Division of Nephrology, Department of Medicine, Taichung Veterans General Hospital, No. 1650 Taiwan Boulevard Sect. 4, Taichung City, 40705 Taiwan; 3 Division of Clinical Toxicology, Department of Medical Toxicology, Taichung Veterans General Hospital, Taichung, Taiwan; 4 Department of Digital Medicine, Taichung Veterans General Hospital, Taichung City, 40705 Taiwan; 5 Department of Applied Mathematics, National Chung Hsing University, Taichung, 402202 Taiwan; 6 Ph.D. Program in Tissue Engineering and Regenerative Medicine, College of Medicine, National Chung Hsing University, Taichung, Taiwan; 7 Department of Applied Life Science and Health, Chia-Nan University of Pharmacy and Science, Tainan, Taiwan; 8 Department of Public Health, College of Public Health, China Medical University, Taichung, Taiwan; 9 Department of Medical Research, China Medical University Hospital, Taichung, Taiwan

**Keywords:** Chronic kidney disease, Machine learning, Infection-related hospitalization, Pre-ESRD, Risk prediction

## Abstract

**Background:**

Patients with advanced chronic kidney disease (CKD), defined as having an estimated glomerular filtration rate (eGFR) < 45 ml/min/1.73 m², are vulnerable to infections leading to hospitalization. Predicting such events using routine clinical data may facilitate early intervention. This study aimed to develop a machine-learning models to predict infection-related hospitalization within 30 days of a nephrology visit.

**Methods:**

We conducted a single center retrospective cohort study of patients in the pre-ESRD pay-for-performance program at Taichung Veterans General Hospital (2010–2022). The primary outcome was infection-related hospitalization within 30 days of the index visit; qualifying admissions required intravenous antibiotic use. We excluded visits from patients < 18 years and those with < 2 nephrology visits in the prior year. Predictor variables included longitudinal laboratory parameters (means and variances within the prior 3 months), comorbidities, and recent medication use. Five machine learning models (XGBoost, random forest, logistic regression, support vector machine, and k-nearest neighbor) were trained and evaluated, with feature importance assessed using Shapley Additive explanations (SHAP).

**Results:**

The analysis included 11,502 index visits from 11,018 patients, with 2,195 infection-related hospitalizations. Among tested models, the XGBoost algorithm achieved the highest predictive performance (AUROC 85.5%, sensitivity 71.3%, specificity 84.8%). The top ten predictors, as ranked by SHAP importance, were recent use of diuretics, serum albumin, Charlson comorbidity index (CCI) excluding renal disease, mean eGFR, corticosteroid use, albumin variance, age, eGFR variance, mean potassium, and mean uric acid. SHAP dependence plots were used to illustrate nonlinear relationships and approximate thresholds associated with increased risk of infection.

**Conclusions:**

Machine learning models using routinely available outpatient data can effectively predict short-term infection-related hospitalization in advanced CKD patients. This approach has the potential to inform individualized surveillance and early intervention strategies in nephrology care.

**Clinical trial number:**

Not applicable.

**Supplementary Information:**

The online version contains supplementary material available at 10.1186/s12879-026-13308-4.

## Introduction

Chronic kidney disease (CKD) affects more than 10% of the global population, making it a major public health concern [1]. Its clinical burden extends beyond progression to end-stage kidney disease, as cardiovascular complications remain the leading cause of death [2]. In addition, hospitalization is common among patients with CKD and is associated with adverse outcomes, including higher risks of readmission, worsening renal function, increased mortality, and diminished quality of life [3].

In the KNOW-CKD study, the overall incidence of hospitalization was 184.96 per 1,000 person-years, with infections ranking second only to circulatory diseases as the most frequent cause [3]. Similarly, the ARIC study reported an incidence rate of infection-related hospitalizations of 23.6 per 1,000 person-years. The risk of infection increased as kidney function declined, with adjusted hazard ratios of 2.55 and 1.48 for patients with eGFR 15–29 and 30–59 ml/min/1.73 m², respectively, compared with those with eGFR ≥ 90 ml/min/1.73 m² [4]. This pattern reflects the impaired immune response associated with kidney dysfunction, which predisposes patients to infections [5]. The most common types of infection include urinary tract and respiratory infections [5], while bloodstream infections and cellulitis are also frequently reported [4].

In patients with advanced CKD, infections not only increase short-term complications but also contribute to long-term adverse outcomes. In the CanPREDDICT cohort, an infectious episode was independently associated with higher risks of cardiovascular ischemia (HR 1.80), congestive heart failure (HR 3.2), and progression to ESKD (HR 1.58) [5]. These findings indicate that infection is more than an acute event; it serves as a pivotal factor driving long-term renal and cardiovascular complications. Preventing infections in this population may therefore have profound implications for slowing CKD progression and reducing cardiovascular burden.

Accurate prediction of infection-related hospitalization in CKD requires robust cohorts and advanced analytic methods. Taiwan’s nationwide pre-ESRD pay-for-performance (P4P) program is well recognized for improving CKD care [6]. Our study was conducted in the largest single center within this program, enrolling more than 10,000 patients with advanced CKD (eGFR < 45 ml/min/1.73 m²). Using routinely collected laboratory, comorbidity, and medication data, we developed machine-learning models to identify patients at high risk of infection-related hospitalization. This work highlights the feasibility of utilizing real-world clinical data to enhance risk stratification in advanced CKD.

## Materials and methods

### Database

This study used data from the pre-end-stage renal disease (pre-ESRD) care database at Taichung Veterans General Hospital (TCVGH), a tertiary medical center in central Taiwan. The pre-ESRD program, supported by Taiwan’s National Health Insurance since 2002, provides structured outpatient care for patients with chronic kidney disease (CKD) and includes those with either an estimated glomerular filtration rate (eGFR) < 45 mL/min/1.73 m² or a urine protein-to-creatinine ratio (UPCR) > 1 g/g. Patients receive regular follow-up, typically every 1 to 3 months, with comprehensive documentation of laboratory data, medication use, comorbidities, and clinical outcomes in the electronic health record.

Although the registry includes patients with isolated proteinuria, we restricted our analysis to those with sustained eGFR < 45 mL/min/1.73 m² to reduce clinical heterogeneity and focus on a high-risk population. All data were de-identified prior to analysis. The study protocol was approved by the Institutional Review Board of TCVGH (IRB No. CE22229B-3).

### Study design and participants

We conducted a retrospective cohort study of patients enrolled in the pre-ESRD program at Taichung Veterans General Hospital between January 1, 2010, and December 31, 2022. Patients became eligible once they were included in the pre-ESRD program and had an estimated glomerular filtration rate (eGFR) < 45 mL/min/1.73 m², confirmed by at least two measurements within a six-month interval. The unit of analysis was the outpatient visit and patients could contribute multiple visits at different time points.

**Fig. 1:**
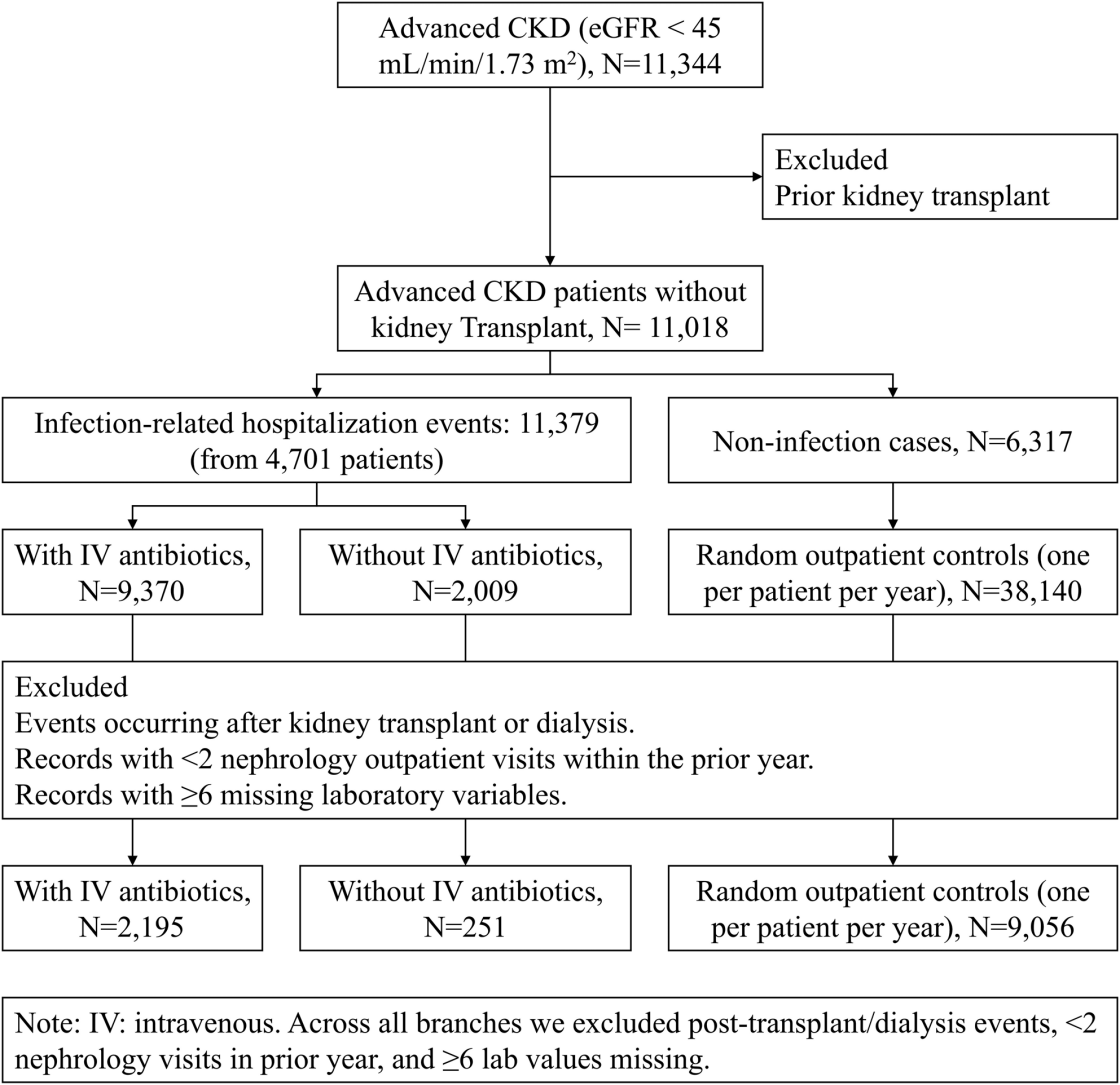
Flow diagram of the study patient and event selection

We applied several exclusion criteria to improve the validity of the analysis. Visits were excluded if the patient was younger than 18 years, had a history of kidney transplantation, or had fewer than two nephrology clinic visits in the year prior to the index visit, which may indicate unstable follow-up. We also excluded records with ≥ 6 missing prespecified laboratory variables. After outcome events were identified, we further excluded any infection-related hospitalizations that occurred after the initiation of chronic dialysis or kidney transplantation, to avoid misclassification due to shifts in treatment modality. After sequential exclusions, as illustrated in Fig. 1, the final analytic dataset included 11,502 visits from 11,018 unique patients.

For each eligible visit, we extracted demographic, laboratory, medication, and comorbidity data from the preceding 3 months to construct features. The primary outcome—hospitalization due to infection—was assessed during the 30-day follow-up period after the visit.

### Outcome definition

The primary outcome was infection-related hospitalization occurring within 30 days following the index outpatient visit. Hospitalizations were identified using ICD-9-CM codes associated with infectious diseases. These encompassed respiratory infections, urinary tract infections, skin and soft tissue infections, joint infections, intra-abdominal and hepatobiliary infections, central nervous system infections, cardiovascular infections, as well as sepsis, bacteremia, and a range of opportunistic or systemic infections including tuberculosis, fungal infections, viral hepatitis, and cytomegalovirus (supplementary Table 1). To improve diagnostic validity, only hospital admissions with intravenous antibiotic use were counted as qualifying events. When multiple hospitalizations occurred within the follow-up window, only the first event was counted. For comparators, we included hospital admissions without intravenous antibiotics and, among patients without infection, randomly sampled one outpatient encounter per patient per calendar year as controls.

### Feature engineering and variable selection

We initially considered multiple clinical predictors across four domains: laboratory tests, urinalysis, medication exposure, and patient characteristics. Variables with a high proportion of missing data, such as HbA1c, UPCR, and hematologic indices, were excluded. The final feature set comprised 34 variables, including age, sex, Charlson Comorbidity Index(CCI) excluding renal disease, presence of pyuria, medication use (ACE inhibitors/ARBs, diuretics, corticosteroids, insulin, cyclophosphamide, calcineurin inhibitors, statins, and metformin), and key laboratory parameters (eGFR, blood urea nitrogen, serum albumin, hemoglobin, sodium, potassium, calcium, phosphorus, uric acid, triglycerides, and LDL cholesterol), each summarized using mean and variance over the preceding three months (supplementary Table 2).

Features with > 20% missing values were excluded. For the remaining variables, mean imputation was used for continuous variables and mode imputation for categorical ones. To assess the potential impact of missing data handling, we performed a sensitivity analysis comparing mean, median, and K-nearest neighbors (KNN) imputation. All continuous features were standardized using z-scores before modeling. Although CKD primary etiology was initially included as a candidate variable, it showed minimal predictive contribution during preliminary analyses and was excluded from the final model, especially considering that comorbidity burden was already captured by the CCI.

### Model development and evaluation

We implemented five supervised learning algorithms to develop prediction models: random forest (RF), extreme gradient boosting (XGBoost), logistic regression (LR), support vector machine (SVM), and k-nearest neighbors (KNN). The dataset was divided into training (80%) and testing (20%) subsets based on unique patient identifiers rather than individual visits. Specifically, all visits from the same patient were assigned exclusively to either the training set or the test set to avoid potential information leakage.

Because the unit of analysis was the outpatient visit and patients could contribute multiple visits, cross-validation during model development was performed using grouped folds based on patient ID, ensuring that repeated visits from the same patient were kept within the same fold. Hyperparameter tuning was conducted using ten-fold cross-validation within the training set.

Model performance was evaluated on the held-out test set using area under the receiver operating characteristic curve (AUROC) as the primary metric. Additional evaluation metrics included sensitivity, specificity, accuracy, and precision. Model selection for downstream interpretability analysis was based on overall discriminative performance and clinical relevance.

### Feature importance analysis

To facilitate model interpretation and identify key clinical variables contributing to prediction, we applied SHapley Additive exPlanations (SHAP) to the selected model. SHAP values quantify the marginal contribution of each feature to the predicted probability at the individual level, enabling both global feature ranking and local interpretability. In addition to overall feature importance, we generated SHAP dependence plots to visualize nonlinear associations between specific predictors and infection risk.

### Statistical analysis

Baseline characteristics were summarized using descriptive statistics. Continuous variables were reported as means with standard deviations (SDs) or medians with interquartile ranges (IQRs), depending on their distribution. Group comparisons between visits with and without subsequent infection-related hospitalization were conducted using independent samples t-tests or Wilcoxon rank-sum tests for continuous variables, and chi-square tests for categorical variables.

Model development and performance evaluation were performed using Python 3.9.13. Machine learning algorithms were implemented with the Scikit-learn (v1.2.2) and XGBoost (v1.7.6) libraries. SHAP (v0.41.0) was used for model interpretability and feature contribution analysis.

Model discrimination was assessed using area under the receiver operating characteristic curve (AUROC), along with sensitivity, specificity, precision, and overall accuracy. The primary outcome was modeled at the visit level, and prediction probabilities were generated for each individual observation in the test set. Feature effects were further evaluated using SHAP summary and dependence plots to explore non-linear associations. All statistical tests were two-sided, and a p-value < 0.05 was considered statistically significant.

To assess the clinical utility of the predictive model, a decision curve analysis (DCA) was performed. The net benefit was calculated by subtracting the proportion of false-positive results from the proportion of true-positive results, weighted by the relative harm of forgoing intervention compared to the harm of unnecessary intervention across a range of threshold probabilities.

## Results

### Characteristics of all study participants

From an initial screening of 11,018 patients, a final analytic cohort of 4,960 unique patients (contributing 11,502 outpatient visits) was included after applying all selection criteria (Fig. 1). Among these visits, 2,195 (19.1%) were followed by an infection-related hospitalization within 30 days. To provide longitudinal context, 1,524 (30.7%) of the 4,960 patients experienced at least one such event over a mean follow-up of 3.33 years, representing an incidence density of 0.09 per person-year.

Table 1 summarizes the clinical characteristics at the time of the outpatient visits. Compared with visits without subsequent infection, those followed by infection-related events were associated with older age (72.9 vs. 69.3 years), higher comorbidity burden (mean CCI: 2.28 vs. 1.10), and lower serum albumin (3.64 vs. 4.13 g/dL). Additionally, more frequent use of diuretics and corticosteroids was observed at these high-risk visits.

**Table 1:** Comparison of clinical characteristics between visits with and without subsequent infection-related hospitalization

	Total Visits (*N* = 11,502)	Visits Without Infection (*N* = 9,307)	Visits With Infection (*N* = 2,195)	*p*-value
**Demographic data**				
Age (years)	70.02 ± 13.56	69.34 ± 13.40	72.92 ± 13.86	< 0.001
Sex (male)	6955 (60.47%)	5607 (60.24%)	1348 (61.41%)	0.3261
**Comorbidity**				
CCI	1.32 ± 1.37	1.10 ± 1.18	2.28 ± 1.67	< 0.001
**CKD stage**				
CKD_1	3095 (26.91%)	2406 (25.85%)	689 (31.39%)	< 0.001
CKD_2	4345 (37.78%)	3604 (38.72%)	741 (33.76%)	
CKD_3	199 (1.73%)	164 (1.76%)	35 (1.59%)	
CKD_4	549 (4.77%)	451 (4.85%)	98 (4.46%)	
CKD_5	3314 (28.81%)	2682 (28.82%)	632 (28.79%)	
**Laboratory**				
BUN_var	132.94 ± 429.45	97.41 ± 367.63	259.42 ± 582.68	< 0.001
BUN_mean	42.58 ± 23.46	40.37 ± 22.44	51.94 ± 25.31	< 0.001
eGFR_var	16.90 ± 62.73	13.24 ± 46.73	30.55 ± 101.21	< 0.001
eGFR_mean	28.20 ± 16.03	29.48 ± 15.85	22.77 ± 15.67	< 0.001
ALB_var	0.05 ± 0.19	0.03 ± 0.10	0.12 ± 0.36	< 0.001
ALB_mean	4.04 ± 0.47	4.13 ± 0.39	3.64 ± 0.55	< 0.001
TG_mean	139.27 ± 93.77	139.36 ± 92.87	138.83 ± 98.14	0.8392
LDL_mean	96.08 ± 32.57	97.33 ± 32.41	89.20 ± 32.63	< 0.001
UA_mean	6.47 ± 1.90	6.35 ± 1.79	7.07 ± 2.28	< 0.001
NA_mean	139.56 ± 3.47	139.89 ± 3.20	138.32 ± 4.10	< 0.001
K_mean	4.51 ± 0.54	4.54 ± 0.52	4.39 ± 0.61	< 0.001
Ca_mean	8.95 ± 0.63	9.02 ± 0.58	8.65 ± 0.71	< 0.001
P_mean	3.97 ± 0.91	3.91 ± 0.84	4.23 ± 1.14	< 0.001
Hgb_mean	11.33 ± 2.20	11.65 ± 2.17	10.13 ± 1.90	< 0.001
Pyurina_latest	1681 (24.58%)	1109 (20.78%)	572 (38.11%)	< 0.001
**Medication**				
Diuretic	3026 (26.31%)	1677 (18.02%)	1349 (61.46%)	< 0.001
Prednisolone	1798 (15.63%)	1005 (10.8%)	793 (36.13%)	< 0.001
insulin	1572 (13.67%)	893 (9.59%)	679 (30.93%)	< 0.001
ACEI/ARB	4784 (41.59%)	3735 (40.13%)	1049 (47.79%)	< 0.001
cyclophosphamide	50 (0.43%)	28 (0.3%)	22 (1.0%)	< 0.001
Calcineurin inhibitors	87 (0.76%)	63 (0.68%)	24 (1.09%)	0.0589
statin	3868 (33.63%)	3092 (33.22%)	776 (35.35%)	0.0607
metformin	246 (2.14%)	198 (2.13%)	48 (2.19%)	0.9276

### Comparison of machine learning models

Among the five machine learning models evaluated, the XGBoost model achieved the highest overall discriminative performance, with an AUROC of 0.855 on the test set. It also demonstrated the most favorable balance of performance metrics, including a sensitivity of 71.28%, a specificity of 84.82% and an accuracy of 82.24%. The RF model yielded a comparable AUROC of 0.86 but had slightly lower accuracy (78.87%). LR (AUROC 0.853) and SVM (AUROC 0.853) models demonstrated moderate performance, while the KNN model had the lowest AUROC (0.753), with markedly reduced sensitivity (42.08%). Given its superior balance of discrimination and sensitivity, the XGBoost model was selected for subsequent SHAP-based interpretation and feature analysis. Full comparative results are summarized in Table 2.

**Table 2:** Performance metrics of machine learning models for infection prediction

	Classifier	Sensitivity	Specificity	Accuracy	AUROC	Precision
Validation	XGBoost	71.28% (3.30%)	84.82% (1.79%)	82.24% (1.72%)	85.52% (2.10%)	52.35% (4.82%)
	RF	78.29% (3.41%)	79.01% (1.94%)	78.87% (1.52%)	86.00% (1.96%)	46.58% (4.06%)
	LR	76.64% (3.47%)	79.28% (1.99%)	78.75% (1.83%)	85.37% (2.33%)	46.39% (4.70%)
	SVM	73.95% (3.70%)	81.52% (2.10%)	80.07% (1.79%)	85.35% (2.27%)	48.39% (5.20%)
	KNN	42.08% (3.17%)	93.57% (0.83%)	83.78% (0.78%)	75.34% (1.60%)	60.40% (4.85%)
Testing	XGBoost	74.72%	84.41%	82.51%	87.76%	53.91%
	RF	81.94%	77.69%	78.52%	87.85%	47.27%
	LR	80.81%	77.41%	78.08%	86.34%	46.61%
	SVM	78.33%	79.78%	79.50%	86.53%	48.60%
	KNN	42.89%	91.68%	82.11%	76.91%	55.72%

RF: random forest; LR: logistic regression; SVM: support vector machine; KNN: k-nearest neighbor; AUROC: area under the receiver operating characteristic curve

In sensitivity analysis using a 12-month feature window, AUROC values remained highly consistent across different imputation strategies (mean, median, and KNN imputation), suggesting that model discrimination was robust to the choice of missing data handling (Supplementary Table 3).

### Selection of feature set and training window

Feature selection was guided by SHAP-based importance ranking. Removing the four predictors with the lowest mean absolute SHAP values (statin, metformin, cyclophosphamide, and calcineurin inhibitor use) reduced the feature set from 25 to 21 variables with minimal impact on AUROC, indicating limited contribution from these variables.

We further compared XGBoost model performance across different training windows (3, 6, 9, and 12 months). Although models trained on more features and longer windows yielded slightly higher AUROCs, the marginal gains were limited. To prioritize clinical applicability and minimize data burden, we selected the 3-month training window and 21-variable model as the preferred configuration for subsequent analyses (Supplementary Fig. 1).

### Clinical utility and practical implications

The decision curve analysis (Supplementary Fig. 2) demonstrated that the machine-learning model provided a higher net benefit compared to both “treat-all” and “treat-none” strategies across a wide range of threshold probabilities. This indicates that using the model to guide clinical decisions for infection-related hospitalization risk stratification is clinically superior to conventional approaches.

### Identification of key predictive features

**Fig. 2:**
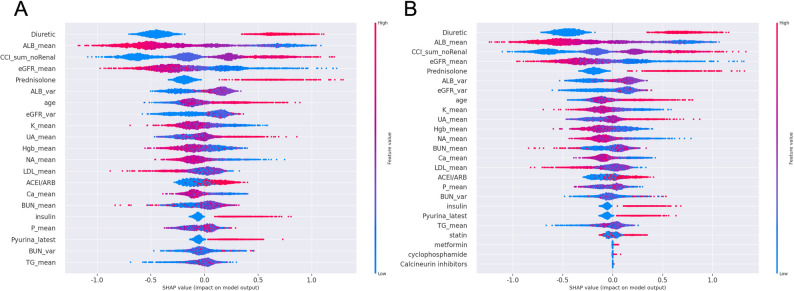
Global feature effects (SHAP summary plots) (**A**) Model using the 21-feature set. (**B**) Model using the 25-feature set. Color encodes raw feature values (red = high, blue = low). Features are ordered by mean absolute SHAP value

SHAP analysis of the final RF model identified the top 21 predictive features contributing to infection-related hospitalization risk. The most influential variables included recent use of diuretics, serum albumin, CCI excluding renal disease, mean eGFR, corticosteroid use, albumin variance, age, eGFR variance, mean potassium, and mean uric acid (Fig. 2).

Regarding the direction of effect, lower mean serum albumin and mean eGFR were both associated with increased predicted risk of infection-related hospitalization. Higher CCI scores and older age were similarly among the strongest risk predictors. The recent use of diuretics and corticosteroids also contributed to higher model-predicted risk. Intra-patient variability in eGFR and serum albumin levels were among the top-ranked features. Additionally, higher uric acid and lower sodium concentrations were positively associated with infection risk prediction.

SHAP-based feature importance demonstrated high consistency between the Random Forest and XGBoost models. In both models, diuretic use, mean serum albumin, CCI excluding renal disease, and prednisolone use were consistently identified as the leading predictors. Despite minor variations in the ranking of secondary variables, fold-specific SHAP analyses confirmed that these primary predictors remained stable across 10-fold grouped cross-validation, as evidenced by their high frequency in the top 10 rankings (Supplementary Table 4).

### Thresholds and risk patterns in laboratory predictors

**Fig. 3:**
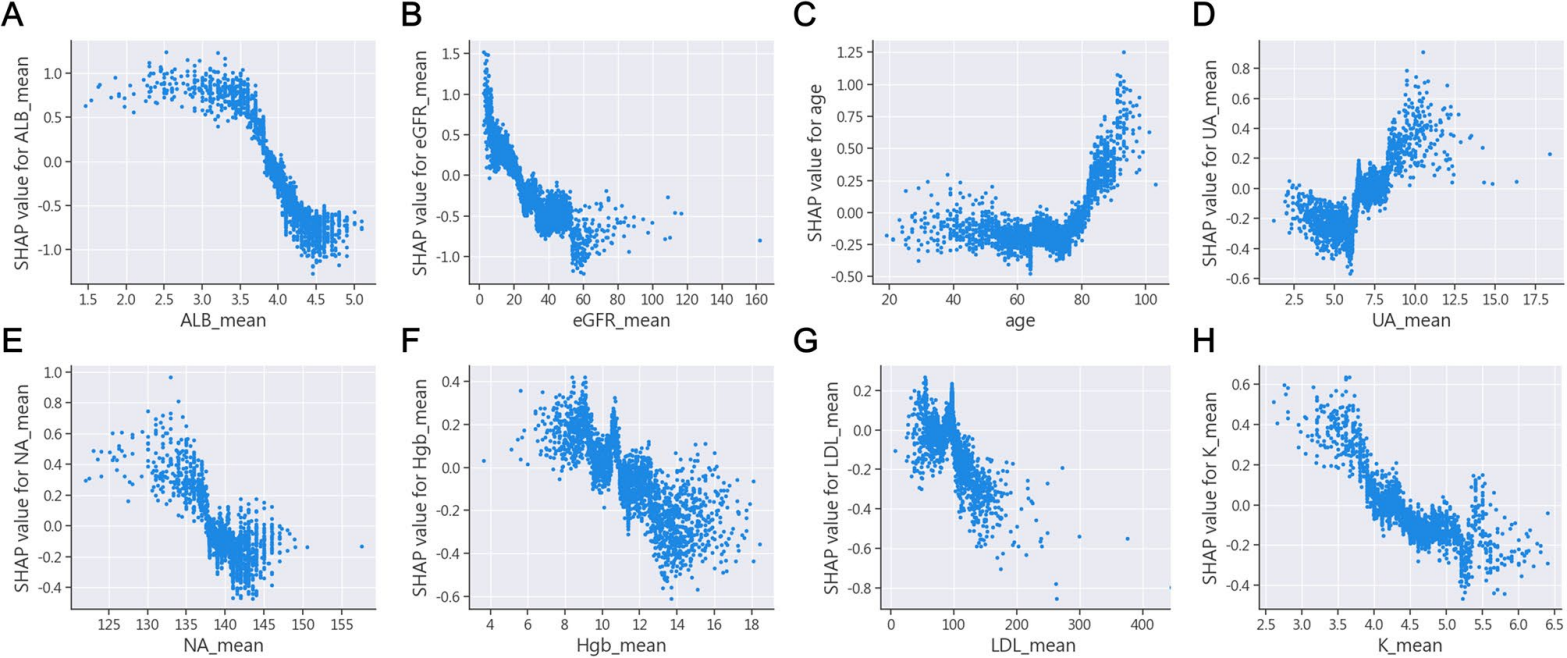
SHAP dependence plots for key predictors (**A**) Albumin, (**B**) eGFR, (**C**) Age, (**D**) Uric acid, (**E**) Sodium, (**F**) Hemoglobin, (**G**) LDL cholesterol, (**H**) Potassium. Each point is a visit; the y-axis is the SHAP value for the corresponding feature

To further elucidate the relationships between laboratory variables and predicted risk, SHAP dependence plots were generated for top-ranked continuous predictors (Fig. 3). These plots revealed predominantly nonlinear relationships, with several clinically meaningful thresholds identified.

Mean serum albumin had the greatest predictive impact, with risk rising sharply below approximately 3.8 g/dL. Mean eGFR was similarly influential, with risk increasing notably below 25 mL/min/1.73 m². Age was positively associated with predicted risk, with a notable increase observed beyond 70 years. Variability in eGFR and albumin was consistently associated with higher risk, indicating that greater fluctuations may reflect underlying clinical instability. Serum uric acid exhibited a gradual increase in risk above 7 mg/dL. Similarly, sodium levels below 138 mmol/L were linked to elevated risk. These findings underscore the utility of dynamic laboratory data in refining infection risk stratification in pre-ESRD care.

## Discussion

In this study of patients with advanced CKD, we developed a machine learning model to predict infection-related hospitalization within 30 days of outpatient visits. The XGBoost model demonstrated the best performance and identified key predictors—including serum albumin, eGFR, comorbidity burden, age, medication use, and laboratory variability. Crucially, decision curve analysis demonstrated the model’s clinical utility, suggesting that its integration into outpatient care can refine risk stratification and optimize resource allocation. By identifying high-risk patients, clinicians can prioritize preventive interventions and intensive monitoring, potentially reducing the infection burden in this vulnerable population.

Immune dysfunction in CKD compromises both innate and adaptive responses, contributing to increased infection susceptibility [7]. Clinical risk factors for infection-related hospitalization in CKD include older age, diabetes, and cardiovascular disease have been shown to increase risk [5]. Advanced CKD (stage IV–V) and ESRD patients predisposes CKD to Clostridium difficile infection [8]. Biochemical and inflammatory markers further refine prediction: lower eGFR [4, 9], albuminuria [4], elevated CRP, neutrophil-to-lymphocyte ratio, leukocytosis [10], anemia and hypoalbuminemia [5] have all been associated with infection risk. Together, these findings underscore the multifactorial nature of infection vulnerability in CKD and the need for comprehensive risk stratification.

Prior machine-learning studies in dialysis populations have mainly addressed all-cause hospitalizations. Fresenius Medical Care (FMC) developed an XGBoost model predicting 12-month hospitalization risk (AUROC 0.81) and another model for 7-day hospitalizations (AUROC 0.78) in dialysis patients [11]. However, no prior studies have specifically focused on infection-related hospitalizations. This gap underscores the novelty of our work, which targets infection as a distinct and clinically important outcome.

Accordingly, we used machine-learning methods to identify influential predictors of infection-related hospitalization. Several of the most influential predictors identified by our model—such as serum albumin, eGFR, comorbidity burden, and age—are consistent with well-established risk factors for infection in patients with advanced CKD. Hypoalbuminemia, a marker of malnutrition and systemic inflammation, is linked to impaired host defenses and higher infection-related morbidity [12]. Lower eGFR independently predicts hospitalization with infection, with graded risk across eGFR categories in the ARIC cohort [4]. CKD features immune dysregulation with increased production—and reduced clearance—of pro-inflammatory cytokines, providing a biologic basis for heightened susceptibility [7]. Age [13] and higher comorbidity burden [14] further compound this risk by contributing to physiological frailty and diminished host defenses.

The model’s identification of recent corticosteroid and diuretic use as key predictors lends further clinical plausibility to its outputs. Even short courses of systemic corticosteroids are associated with immunosuppressive effects and substantially higher risks of common infections—including respiratory tract infections and cellulitis—in population-based studies [15]. Diuretic use, on the other hand, often reflects the management of fluid overload or heart failure in patients with cardiorenal syndrome, thereby serving as a marker of hemodynamic instability [16]. Taken together, these medication-related signals suggest that the model captures not only underlying patient characteristics but also current disease activity and treatment response.

Our model highlighted the prognostic value of laboratory variability, with fluctuations in eGFR [17] and albumin [18] shown to predict adverse outcomes in CKD and dialysis cohorts, reflecting episodes of nutritional decline, inflammation, or hemodynamic instability. These dynamic patterns are clinically meaningful, as they capture patient trajectories rather than snapshots, aligning with real-world instability that often precedes acute events. Similarly, hyponatremia [19] demonstrates a relationship with mortality in CKD, while higher uric acid [20] correlates with frailty in older adults, serving as a marker of systemic metabolic and inflammatory stress.

This study utilizes one of the largest advanced-CKD cohorts within Taiwan’s pre-ESRD P4P program [6], where regimented laboratory schedules enabled consistent 3-month, time-windowed feature construction and minimized measurement heterogeneity. Outcome ascertainment required concomitant antibiotic therapy at hospitalization, improving specificity for infection-related admissions. SHAP dependence plots (Fig. 3) reveal curved risk patterns with practical thresholds (e.g., low albumin, dysnatremia, greater eGFR variability), suggesting actionable thresholds. Together, these findings support translation into a prediction score—built from short-horizon outpatient labs plus a few medication indicators (e.g., diuretics, corticosteroids)—with practical bedside utility for further surveillance and early intervention.

Several limitations warrant consideration. First, this single-center study from a tertiary medical center may limit generalizability; external validation in independent cohorts and care models is needed to assess transportability. Second, although internal performance was strong, overfitting remains a risk; we used ten-fold cross-validation, conservative feature selection to reduce dimensionality, and an ensemble learner (XGBoost), and we preserved a strictly held-out test set for final evaluation, but prospective, multicenter validation is still required. Third, predictor availability was constrained: lifestyle and anthropometric variables (e.g., smoking status, body mass index) and key inflammatory markers—C-reactive protein (CRP), neutrophil-to-lymphocyte ratio (NLR), and leukocyte count—were not routinely captured in a time-aligned manner in the pre-ESRD database and were therefore excluded; not including them may modestly lower accuracy and obscure inflammation-related signals. Finally, by defining the outcome as infection-related hospitalization with concurrent antibiotic use, we improved specificity but may have missed cases managed without admission or without antibiotics.

## Conclusion

Using routinely collected outpatient data, we established a machine-learning model capable of predicting short-term infection-related hospitalization in advanced CKD. The model demonstrated robust performance and identified predictors consistent with clinical plausibility. These results suggest that machine-learning–based risk stratification could augment routine nephrology care, while underscoring the need for external validation in other populations and healthcare systems.

## Supplementary Information

Below is the link to the electronic supplementary material.

Supplementary Material 1

Supplementary Material 2: Figure S1. AUROC by feature time window and feature set

Supplementary Material 3

## Data Availability

The data supporting the findings of this study are available from the pre-ESRD database of Taichung Veterans General Hospital. Data were de-identified prior to analysis. Due to privacy restrictions, these data are not publicly available but may be requested from the corresponding author, contingent upon IRB approval.

## Abbreviations

ACE: Angiotensin-converting enzyme
ARB: Angiotensin receptor blocker
AUROC: Area under the receiver operating characteristic curve
CCI: Charlson Comorbidity Index
CKD: Chronic kidney disease
CRP: C-reactive protein
eGFR: Estimated glomerular filtration rate
ESRD: End-stage renal disease
HbA1c: Hemoglobin A1c
HR: Hazard ratio
KNN: K-nearest neighbors
LDL: Low-density lipoprotein
LR: Logistic regression
NLR: Neutrophil-to-lymphocyte ratio
RF: Random forest
SHAP: SHapley Additive exPlanations
SVM: Support vector machine
TCVGH: Taichung Veterans General Hospital
UPCR: Urine protein-to-creatinine ratio
XGBoost: Extreme gradient boosting
