# Supplementary material for: Machine-learning prediction of 30-day infection-related hospitalization in advanced CKD
Source: BMC Infect Dis. 2026 May 30;26:1429. doi: 10.1186/s12879-026-13308-4 (PMC13439995; doi:10.1186/s12879-026-13308-4)
Supplement: Supplementary file 1 [file 12879_2026_13308_MOESM1_ESM.docx]

Supplementary Table 1. ICD-9-CM and ICD-10-CM Codes Used to Define Infection-Related Hospitalizations.

| **Respiratory tract** |  |  |
| --- | --- | --- |
| Acute respiratory infections |  |  |
| ICD9 | 460–466 | Acute Respiratory Infections |
|  | 460 | Acute nasopharyngitis |
|  | 461 | Acute sinusitis |
|  | 462 | Acute pharyngitis |
|  | 463 | Acute tonsillitis |
|  | 464 | Acute laryngitis and tracheitis |
|  | 465 | Acute upper respiratory infections of multiple or unspecified sites |
|  | 466 | Acute bronchitis and bronchiolitis |
| ICD10 | J00 | Acute nasopharyngitis |
|  | J01 | Acute sinusitis |
|  | J02 | Acute pharyngitis |
|  | J03 | Acute tonsillitis |
|  | J04 | Acute laryngitis and tracheitis |
|  | J05 | Acute obstructive laryngitis [croup] and epiglottitis |
|  | J06 | Acute upper respiratory infections of multiple and unspecified sites |
|  | J20 | Acute bronchitis |
|  | J21 | Acute bronchiolitis |
| Pneumonia and influenza |  |  |
| ICD9 | 480–488 | Pneumonia And Influenza |
|  | 480 | Viral pneumonia |
|  | 481 | Pneumococcal pneumonia |
|  | 482 | Other bacterial pneumonia |
|  | 483 | Pneumonia due to other specified organism |
|  | 484 | Pneumonia in infectious diseases classified elsewhere |
|  | 485 | Bronchopneumonia, organism unspecified |
|  | 486 | Pneumonia, organism unspecified |
|  | 487 | Influenza |
|  | 488 | Influenza due to certain identified influenza viruses |
| ICD10 | J09 | Influenza due to certain identified influenza viruses |
|  | J10 | Influenza due to other identified influenza virus |
|  | J11 | Influenza due to unidentified influenza virus |
|  | J12 | Viral pneumonia, not elsewhere classified |
|  | J13 | Pneumonia due to Streptococcus pneumoniae |
|  | J14 | Pneumonia due to Hemophilus influenzae |
|  | J15 | Bacterial pneumonia, not elsewhere classified |
|  | J16 | Pneumonia due to other infectious organisms, not elsewhere classified |
|  | J17 | Pneumonia in diseases classified elsewhere |
|  | J18.0 | Bronchopneumonia, unspecified organism |
|  | J18.1 | Lobar pneumonia, unspecified organism |
|  | J18.8 | Other pneumonia, unspecified organism |
|  | J18.9 | Pneumonia, unspecified organism |
|  | A22.1 | Pulmonary anthrax |
|  | A37.91 | Whooping cough, unspecified species with pneumonia |
|  | A48.1 | Legionnaires' disease |
|  | B25.0 | Cytomegaloviral pneumonitis |
|  | B44.0 | Invasive pulmonary aspergillosis |
| Empyema |  |  |
| ICD9 | 510 | Empyema |
| ICD10 | J86 | Pyothorax |
| Abscess of lung and mediastinum |  |  |
| ICD9 | 513 | Abscess of lung and mediastinum |
| ICD10 | J85 | Abscess of lung and mediastinum |
| **Urinary tract** |  |  |
| Kidney |  |  |
| ICD9 | 590 | Infections of kidney |
| ICD10 | N10 | Acute pyelonephritis |
|  | N11 | Chronic tubulo-interstitial nephritis |
|  | N12 | Tubulo-interstitial nephritis, not specified as acute or chronic |
|  | N13.6 | Pyonephrosis |
|  | N15.1 | Renal and perinephric abscess |
|  | N15.9 | Renal tubulo-interstitial disease, unspecified |
|  | N16 | Renal tubulo-interstitial disorders in diseases |
|  | N28.84 | Pyelitis cystica |
|  | N28.85 | Pyeloureteritiscystica |
|  | N28.86 | Ureteritiscystica |
| Cystitis |  |  |
| ICD9 | 595 | Cystitis |
| ICD10 | N30 | Cystitis |
| **Skin, joint and soft tissues** |  |  |
| Skin and soft tissue |  |  |
| ICD9 | 680-686 | Infections Of Skin And Subcutaneous Tissue |
|  | 680 | Carbuncle and furuncle |
|  | 681 | Cellulitis and abscess of finger and toe |
|  | 682 | Other cellulitis and abscess |
|  | 683 | Acute lymphadenitis |
|  | 684 | Impetigo |
|  | 685 | Pilonidal cyst |
|  | 686 | Other local infections of skin and subcutaneous tissue |
| ICD10 | L01 | Impetigo |
|  | L02 | Cutaneous abscess, furuncle and carbuncle |
|  | L03 | Cellulitis and acute lymphangitis |
|  | L04 | Acute lymphadenitis |
|  | L05 | Pilonidal cyst and sinus |
|  | L08.0 | Pyoderma |
|  | L08.8 | Other specified local infections of the skin and subcutaneous tissue |
|  | L08.9 | Local infection of the skin and subcutaneous |
|  | L88 | Pyoderma gangrenosum |
|  | L92.8 | Other granulomatous disorders of the skin and subcutaneous tissue |
|  | L98.0 | Pyogenic granuloma |
|  | L98.3 | Eosinophilic cellulitis [Wells] |
|  | B78.1 | Cutaneous strongyloidiasis |
|  | E83.2 | Disorders of zinc metabolism |
|  | K12.2 | Cellulitis and abscess of mouth |
| Joint |  |  |
| ICD9 | 711 | Arthropathy associated with infections |
| ICD10 | M00 | Pyogenic arthritis |
|  | M01 | Direct infections of joint in infectious and parasitic diseases classified elsewhere |
|  | M02 | Postinfective and reactive arthropathies |
|  | M35.2 | Behcet's syndrome |
| **Gastrointestinal tract** |  |  |
| Abscess of salivary gland |  |  |
| ICD9 | 527.3 | Abscess of salivary gland |
| ICD10 | K11.3 | Abscess of salivary gland |
| Cellulitis and abscess of oral softtissues |  |  |
| ICD9 | 528.3 | Cellulitis and abscess of oral soft tissues |
| ICD10 | K12.2 | Cellulitis and abscess of mouth |
| Abscess of anal and rectal regions |  |  |
| ICD9 | 566 | Abscess of anal and rectal regions |
| ICD10 | K61 | Abscess of anal and rectal regions |
| Peritonitis and retroperitoneal infections |  |  |
| ICD9 | 567 | Peritonitis and retroperitoneal infections |
| ICD10 | K65 | Peritonitis |
|  | K67 | Disorders of peritoneum in infectious diseases classified elsewhere |
|  | K68.11 | Postprocedural retroperitoneal abscess |
|  | K68.19 | Other retroperitoneal abscess |
|  | K68.9 | Other disorders of retroperitoneum |
| Abscess of liver |  |  |
| ICD9 | 572.0 | Abscess of liver |
| ICD10 | K75.0 | Abscess of liver |
| Hepatitis in viral diseases classified elsewhere |  |  |
| ICD9 | 573.1 | Hepatitis in viral diseases classified elsewhere |
| ICD10 | K77 | Liver disorders in diseases classified elsewhere |
| Acute cholecystitis |  |  |
| ICD9 | 575.0 | Acute cholecystitis |
| ICD10 | K81.0 | Acute cholecystitis |
| **Central nervous system & ENT** |  |  |
| ICD9 | 320 | Bacterial meningitis |
|  | 321 | Meningitis due to other organisms |
|  | 322 | Meningitis of unspecified cause |
|  | 323 | Encephalitis myelitis and encephalomyelitis |
|  | 324 | Intracranial and intraspinal abscess |
|  | 325 | Phlebitis and thrombophlebitis of intracranial venous sinuses |
|  | 326 | Late effects of intracranial abscess or pyogenic infection |
| ICD10 | G00 | Bacterial meningitis, not elsewhere classified |
|  | G01 | Meningitis in bacterial diseases classified elsewhere |
|  | G02 | Meningitis in other infectious and parasitic diseases classified elsewhere |
|  | G03.0 | Nonpyogenic meningitis |
|  | G03.1 | Chronic meningitis |
|  | G03.8 | Meningitis due to other specified causes |
|  | G03.9 | Meningitis, unspecified |
|  | G04.0 | Acute disseminated encephalitis and encephalomyelitis (ADEM) |
|  | G04.2 | Bacterial meningoencephalitis and meningomyelitis, not elsewhere classified |
|  | G04.3 | Acute necrotizing hemorrhagic encephalopathy |
|  | G04.8 | Other encephalitis, myelitis and encephalomyelitis |
|  | G04.9 | Encephalitis, myelitis and encephalomyelitis, unspecified |
|  | G05 | Encephalitis, myelitis and encephalomyelitis in diseases classified elsewhere |
|  | G06 | Intracranial and intraspinal abscess and granuloma |
|  | G07 | Intracranial and intraspinal abscess and granuloma in diseases classified elsewhere |
|  | G08 | Intracranial and intraspinal phlebitis and thrombophlebitis |
|  | G09 | Sequelae of inflammatory diseases of central nervous system |
|  | G37.3 | Acute transverse myelitis in demyelinating disease of central nervous system |
|  | G37.4 | Subacute necrotizing myelitis of central nervous system |
|  | G92 | Toxic encephalopathy |
|  | B45.1 | Cerebral cryptococcosis |
| **CNS** |  |  |
| Suppurative and unspecified otitis media |  |  |
| ICD9 | 382 | Suppurative and unspecified otitis media |
| ICD10 | H66, | Suppurative and unspecified otitis media |
|  | H67 | Otitis media in diseases classified elsewhere |
| Mastoiditis and related conditions |  |  |
| ICD9 | 383 | Mastoiditis and related conditions |
| ICD10 | H70 | Mastoiditis and related conditions |
|  | H75 | Other disorders of middle ear and mastoid in diseases classified elsewhere |
|  | H95.0 | Recurrent cholesteatoma of postmastoidectomy cavity |
|  | H95.1 | Other disorders of ear and mastoid process following mastoidectomy |
| **Cardiovascular system** |  |  |
| Acute pericarditis |  |  |
| ICD9 | 420 | Acute pericarditis |
| ICD10 | I30 | Acute pericarditis |
|  | I32 | Pericarditis in diseases classified elsewhere |
| Acute and subacute endocarditis |  |  |
| ICD9 | 421 | Acute and subacute endocarditis |
| ICD10 | I33 | Acute and subacute endocarditis |
|  | I39 | Endocarditis and heart valve disorders in diseases classified elsewhere |
| Acute myocarditis |  |  |
| ICD9 | 422 | Acute myocarditis |
| ICD10 | I40 | Acute myocarditis |
|  | I41 | Myocarditis in diseases classified elsewhere |
| **Bacteriamia/septicaemia** |  |  |
| ICD9 | 038 | Septicemia |
|  | 790.7 | Bacteremia |
| ICD10 | A40 | Streptococcal sepsis |
|  | A41 | Other sepsis |
|  | R65.1 | Systemic inflammatory response syndrome (SIRS) of non-infectious origin |
|  | R65.20 | Severe sepsis without septic shock |
|  | R78.81 | Bacteremia |
| **Salmonella infections** |  |  |
| ICD9 | 003 | Other salmonella infections |
| ICD10 | A02 | Other salmonella infections |
| **Opportunistic infection** |  |  |
| Pulmonary tuberculosis |  |  |
| ICD9 | 010 | Primary tuberculous infection |
|  | 011 | Pulmonary tuberculosis |
|  | 012 | Other respiratory tuberculosis |
|  | 018 | Miliary tuberculosis |
| ICD10 | A15 | Respiratory tuberculosis |
|  | A19 | Miliary tuberculosis |
| Extra-PTB |  |  |
| ICD9 | 013 | Tuberculosis of meninges and central nervous system |
|  | 014 | Tuberculosis of intestines peritoneum and mesenteric glands |
|  | 015 | Tuberculosis of bones and joints |
|  | 016 | Tuberculosis of genitourinary system |
|  | 017 | Tuberculosis of other organs |
| ICD10 | A17 | Tuberculosis of nervous system |
|  | A18 | Tuberculosis of other organs |
| Atypical mycobacteria |  |  |
| ICD9 | 031 | Diseases due to other mycobacteria |
| ICD10 | A31 | Infection due to other mycobacteria |
| Cryptococcosis |  |  |
| ICD9 | 117.5 | Cryptococcosis |
| ICD10 | B45.0 | Pulmonary cryptococcosis |
|  | B45.2 | Cutaneous cryptococcosis |
|  | B45.3 | Osseous cryptococcosis |
|  | B45.7 | Disseminated cryptococcosis |
|  | B45.8 | Other forms of cryptococcosis |
|  | B45.9 | Cryptococcosis, unspecified |
| Aspergillosis |  |  |
| ICD9 | 117.3 | Aspergillosis |
| ICD10 | B44.1 | Other pulmonary aspergillosis |
|  | B44.2 | Tonsillar aspergillosis |
|  | B44.7 | Disseminated aspergillosis |
|  | B44.89 | Other forms of aspergillosis |
|  | B44.9 | Aspergillosis, unspecified |
|  | B48.4 | Penicillosis |
| Histoplasmosis |  |  |
| ICD9 | 115 | Histoplasmosis |
| ICD10 | B39 | Histoplasmosis |
| Listeriosis |  |  |
| ICD9 | 027.0 | Listeriosis |
| ICD10 | A32 | Listeriosis |
| Leishmaniasis |  |  |
| ICD9 | 085 | Leishmaniasis |
| ICD10 | B55 | Leishmaniasis |
| Pneumocystis jiroveci pneumonia |  |  |
| ICD9 | 136.3 | Pneumocystosis |
| ICD10 | B59 | Pneumocystosis |
| **Viral infection** |  |  |
| Herpes zoster |  |  |
| ICD9 | 053 | Herpes zoster |
| ICD10 | B02 | Zoster [herpes zoster] |
| Hepatitis B |  |  |
| ICD9 | 070.2 | Mumps encephalitis |
|  | 070.3 | Mumps pancreatitis |
| ICD10 | B16 | Acute hepatitis B |
|  | B18.0 | Chronic viral hepatitis B with delta-agent |
|  | B18.1 | Chronic viral hepatitis B without delta-agent |
|  | B19.1 | Unspecified viral hepatitis B |
| Hepatitis C |  |  |
| ICD9 | 070.7 | Unspecified viral hepatitis c |
| ICD10 | B19.2 | Unspecified viral hepatitis C |
| Viremia |  |  |
| ICD9 | 790.8 | Viremia, unspecified |
| ICD10 | B34.9 | Viral infection, unspecified |
| CMV |  |  |
| ICD9 | 078.5 | Cytomegaloviral disease |
| ICD10 | B25.8 | Other cytomegaloviral diseases |
|  | B25.9 | Cytomegaloviral disease, unspecified |

Supplementary table 2. List of Candidate Variables Considered for Infection Prediction.

| Category | Number of characteristics (total 34) |
| --- | --- |
| Laboratory tests | 22 (11x2) |
| Serum: eGFR, BUN, Alb, Na, K, Ca, P, Hgb, TG, LDL, UA | mean, variance within 3 months |
| Urine: pyuria or not | 1 |
| Medications: ACEI/ARB, prednisolone, cyclophosphamide, calcineurin inhibitors, statin, diuretic, insulin, metformin | 8 |
| Past history: Age, sex, CCI | 3 |

Supplementary Table 3. Sensitivity Analysis of Imputation Strategies on Model Performance Using a 12-Month Feature Window.

| **Feature set** | **Model** | **Mean** | **Median** | **KNN** |
| --- | --- | --- | --- | --- |
| **21 features** | XGBoost | 87.99% | 88.02% | 87.69% |
|  | Random Forest | 88.14% | 88.26% | 88.14% |
|  | Logistic Regression | 86.82% | 86.82% | 86.75% |
|  | SVM | 86.86% | 86.86% | 86.77% |
|  | KNN | 77.08% | 76.76% | 76.68% |
| **25 features** | XGBoost | 88.11% | 88.26% | 87.99% |
|  | Random Forest | 88.42% | 88.41% | 88.28% |
|  | Logistic Regression | 86.76% | 86.76% | 86.70% |
|  | SVM | 86.87% | 86.87% | 86.80% |
|  | KNN | 76.19% | 76.29% | 75.81% |

Sensitivity analysis was conducted using a 12-month feature window to compare mean, median, and K-nearest neighbors (KNN) imputation methods across five machine learning models.

Supplementary Table 4. Stability of SHAP-based feature importance rankings across 10-fold grouped cross-validation for the Random Forest and XGBoost models.

| Random Forest | | | |
| --- | --- | --- | --- |
| feature | mean_rank | std_rank | top10_frequency |
| Diuretic | 1 | 0 | 10 |
| ALB_mean | 2 | 0 | 10 |
| CCI_sum_noRenal | 3 | 0 | 10 |
| Hgb_mean | 4.2 | 0.42 | 10 |
| Prednisolone | 4.8 | 0.42 | 10 |
| Ca_mean | 6.4 | 0.52 | 10 |
| eGFR_mean | 6.6 | 0.52 | 10 |
| BUN_mean | 8.3 | 0.48 | 10 |
| NA_mean | 9.2 | 1.03 | 9 |
| insulin | 10 | 1.05 | 7 |
| XGBoost | | | |
| feature | mean_rank | std_rank | top10_frequency |
| Diuretic | 1.1 | 0.32 | 10 |
| ALB_mean | 1.9 | 0.32 | 10 |
| CCI_sum_noRenal | 3 | 0 | 10 |
| eGFR_mean | 4 | 0 | 10 |
| Prednisolone | 5 | 0 | 10 |
| ALB_var | 6.7 | 0.82 | 10 |
| age | 7.2 | 1.23 | 10 |
| eGFR_var | 8.4 | 1.51 | 8 |
| Hgb_mean | 10.6 | 2.63 | 5 |
| UA_mean | 10.8 | 1.69 | 5 |

Abbreviations: mean_rank, the average ranking of the feature across 10 folds (1 is the most important); std_rank, the standard deviation of the ranking; top10_frequency, the number of times the feature appeared among the top 10 most important predictors across the 10 folds.
